# “Like a ticking time bomb”: the persistence of trauma in the HIV diagnosis experience among black men who have sex with men in New York City

**DOI:** 10.1186/s12889-020-09342-9

**Published:** 2020-08-17

**Authors:** Ofole Mgbako, Ellen Benoit, Nishanth S. Iyengar, Christopher Kuhner, Dustin Brinker, Dustin T. Duncan

**Affiliations:** 1 Division of Infectious Disease, Department of Internal Medicine, Columbia University Medical Center, 630 W. 168th St., P&S Box 82, New York, NY 10032 USA; 2 North Jersey Community Research Initiative, Newark, NJ USA; 3 NYU Grossman School of Medicine, New York, NY USA; 4 Columbia Spatial Epidemiology Lab, Social and Spatial Epidemiology Unit, Department of Epidemiology, Columbia University Mailman School of Public Health, New York, USA

**Keywords:** Black, MSM, African-American, HIV, Trauma

## Abstract

**Background:**

Black men who have sex with men (MSM) are disproportionately affected by HIV compared to almost every other demographic group in the country and have worse outcomes along the care continuum. Diagnosis is a critical juncture. This study aims to explore the impact and meaning of an HIV diagnosis for Black MSM, and how this has changed over time, both for the individual’s experience living with HIV as well as for Black MSM in general.

**Methods:**

From 2017 to 2018, we conducted in-depth interviews with 16 black MSM living with HIV in New York City diagnosed between 1985 and 2016.

**Results:**

Inductive analysis of the qualitative data allowed three major themes to emerge: diagnosis trauma, lack of patient -centeredness in the healthcare system, and acceptance of HIV diagnosis over time.

**Conclusions:**

This small pilot study signals that an HIV diagnosis experience possibly remains traumatic for black MSM even in the era of highly effective ART, and they often perceive a lack of patient-centeredness in the delivery of a new diagnosis. This has persisted over time. In most cases, black MSM in our sample overcame this trauma due to self-motivation, social support and seeking out and fostering trusting relationships with their HIV provider and the healthcare system.

## Background

Black or African American men who have sex with men (MSM) are disproportionately affected by HIV compared to nearly every other demographic group in the country, comprising 26% of all new HIV diagnoses in 2017 [1]. Furthermore, compared to white MSM, black MSM have worse outcomes at each stage along the HIV care continuum, a notable trend in cities across the country [2, 3]. Diagnosis is an especially critical juncture of the HIV care continuum that marks the transition from HIV-negative to HIV-positive status. The HIV diagnosis experience heavily influences whether black MSM newly diagnosed with HIV will link to care, remain adherent to antiretroviral therapy (ART), and prioritize ongoing clinical care to achieve and sustain viral load suppression [4].

An unpleasant HIV diagnosis experience has always carried particular resonance for racial, gender and sexual minorities. A 1997 qualitative study of women of color newly diagnosed with HIV showed that receiving an HIV diagnosis was a traumatic event which represented a threat to their lives, leading to misery, substance use, and destabilization of relationships [5]. A more recent study from the current era of highly active ART (HAART) among high-risk black/Latino heterosexual adults showed similar problems accepting and internalizing an HIV diagnosis due to fear of stigma and loss of relationships [6]. A more in-depth exploration of the HIV diagnosis experience among black MSM and how it has changed over time is necessary to understand the important factors and psychological processes to examine at time of diagnosis for one of the country’s most at-risk groups for HIV.

For black MSM, contextual factors such as medical mistrust, diagnosis denial, HIV-related stigma, and previous experiences with discrimination come into play before and after diagnosis. A 2018 quantitative study among a cohort of black MSM pre- and post-HIV diagnosis found an association between lack of engagement in HIV care and anticipated HIV-related stigma [7]. A qualitative study by Carey et al. that explored barriers to care engagement among black and Latino MSM in New York City (NYC) showed a delay in ART initiation due to denial of diagnosis [8]. Freeman et al. found that HIV care experiences among mostly black and Latino MSM are viewed through the lens of past and present structural racism manifested in segregated communities, poverty and unequal distribution of resources. As a result, patients often felt distrustful of institutions and providers, and felt excluded from the decision-making process [9]. Indeed, multiple studies have established that black MSM expect their experience with HIV care to be accompanied by racism, homophobia, and HIV-related stigma and discrimination [10–13]. However, few studies have explored the diagnosis experience itself in an in-depth fashion.

Our focus in this paper is limited to diagnosis experiences, although the data gathered was part of a broader formative study examining black MSM experiences along the HIV care continuum. Funded by a small university grant, the study was conducted to establish pilot data for subsequent research. Our analysis builds on previous understanding of the HIV diagnosis experience in the literature from the perspective of black MSM and explores the impact and meaning of an HIV diagnosis in relation to the many biomedical advances in HIV care over the last three decades. As improved care coordination and HAART have increased the life expectancy of people living with HIV/AIDS, and public health efforts have been put in place to reclassify HIV as a manageable chronic illness, it is important to understand how an HIV diagnosis is perceived by black MSM in the context of structural racism and homophobia, and HIV-related stigma. It is also important to understand how this has changed over time from the pre-HAART to the post-HAART era, both for the individual’s experience living with HIV as well as for black MSM in general. The way in which a new diagnosis is delivered, the issues explored and emphasized by health care providers, the perception of HIV itself and of the patient-provider interaction at diagnosis all inform ongoing engagement with the healthcare system and other risk behaviors [14]. In this qualitative study, we analyzed experiences related by black MSM diagnosed between 1985 and 2016, examining how perceptions changed over time.

## Methods

### Participants and eligibility

Between 2017 and 2018, we recruited a sample of black or African-American MSM (*N* = 16) with a self-reported diagnosis of HIV for a brief demographic survey and an in-depth semi-structured interview. We recruited participants until we attained data saturation. In order to have a diverse sample, we employed in-person recruitment at various local organizations and via flyers in multiple locations throughout NYC, including the Bellevue Virology Clinic, Callen Lorde Community Health Center, Gay Men’s Health Crisis, Brooklyn Men Konnect and the LGBT Center, as well as at Brooklyn and Harlem Pride events. Advertisements were also placed on Twitter.com, Craigslist.com, and in the local newspaper Caribbean Life. Eligibility criteria included: age 18–65 years, self-identifying as black, self-identifying as male, self-reported HIV, able to understand and read English, willing and able to provide informed consent, living in one of the five boroughs of NYC, and having had sexual intercourse (oral or anal) with another man in the last 12 months.

### Survey

The quantitative survey captured basic demographic information including age, race/ethnicity, sexual orientation, level of education, income, housing, employment, history of incarceration and health insurance status. Additional file 1 shows this in more detail (see Additional file 1). We then asked questions about sexual practices, sexually transmitted diseases and substance use. We collected information on HIV diagnosis, including date tested positive, to further contextualize the diagnosis experience. We also collected information about linkage to care, retention in care, adherence to antiretroviral medications, and viral load suppression. Participants filled out the paper questionnaire independently and a member of the research team was available to answer questions. Responses were recorded and stored in REDCap.

### Qualitative interview

To obtain a more complete understanding of the care experiences captured in the survey, particularly the diagnosis experience, we conducted one-on-one in-depth semi-structured interviews. Interviews were conducted in English by three trained qualitative researchers – OM, CK, and DB – and took place from January 1, 2018 to June 30, 2018. All interviews were conducted in a private office in a public hospital in NYC. Additional file 2 shows that the interview guide was organized by the HIV care continuum and included in-depth questions about the diagnosis experience, barriers to linkage to and retention in care, barriers to medication initiation and adherence, and the role of racial, sexual and cultural identity in HIV care [see Additional file 2]. This guide allowed for exploration of emergent themes as they arose. Interviewers were all trained in qualitative interviewing by a PhD in behavioral science research with a focus on qualitative methods. Each interview lasted between 90 and 120 min, was audio-recorded, and then transcribed verbatim by a professional transcription service. At the conclusion of the interview, each participant was compensated $40.

### Analysis

We computed descriptive statistics on the survey responses. Using Dedoose software, the first author and a trained qualitative analyst (EB) coded three interviews independently and then reconciled their codes to develop a code book and establish inter-rater reliability. Three authors – OM, CK, DB – then applied those codes to the entire sample of interviews. We grouped coded segments of text into categories based on content similarity and then we reread the coded narrative descriptions of the diagnosis experience using an inductive process, which was guided by the research questions but allowed themes to emerge with no pre-existing framework [15, 16]. We followed COREQ (consolidated criteria for reporting qualitative research) guidelines on the reporting for qualitative research [17]. Additional file 3 shows the COREQ guideline (see Additional file 3).

## Results

### Demographics

Table 1 shows baseline demographic characteristics and information related to the HIV care continuum of the sample (*n* = 16). The ages ranged from 25 to 55 years old (mean 42, ± 9.6). All participants identified as black/African-American. Most participants (75%) reported being single, half (50%) lived in the Bronx, and most (94%) identified as gay/homosexual. Almost half (44%) of participants completed some college, about three-quarters (75%) were employed for wages or self-employed, and most (88%) had stable housing in the previous year. Over half (56%) of participants reported an annual income of less than $20,000. Two (12%) participants reported a history of incarceration and the majority (94%) of participants had health insurance.

**Table 1:** Sample demographics and HIV care continuum characteristics

Factor	N (%)
Race/Ethnicity	
Black/African American	16 (100)
Hispanic/Latino	2 (13)
White/mixed	1 (6.3)
NYC borough	
Manhattan	5 (3.1)
Bronx	8 (50)
Brooklyn	1 (6.3)
Queens	1 (6.3)
Staten Island	1 (6.3)
Sexual identity	
Gay/homosexual	15 (94)
Bisexual	1 (6.3)
Education	
Some high school	1 (6.3)
Some college	7 (44)
Degree from vocational/technical school	1 (6.3)
Associate degree	3 (19)
Bachelor’s degree	2 (13)
Advanced degree	2 (13)
Employment status	
Employed for wages	10 (63)
Self-employed	2 (13)
Unemployed and looking for work	3 (19)
Unemployed and not looking for work	1 (6.3)
Housing status	
Currently housed	14 (88)
Currently homeless	2 (13)
Homeless in the past 12 months	3 (19)
Annual income	
< $20,000	9 (56)
$20,000 – $40,000	4 (25)
$40,000 – $60,000	1 (6.3)
> $100,000	2 (13)
Health insurance	
Yes	15 (94)
No	1 (6.3)
Relationship status	
Single	12 (75)
Partnered	2 (13)
Married	2 (13)
STI diagnosis in the past year	
Yes	2 (13)
Syphilis	1 (6.3)
Gonorrhea	1 (6.3)
No	14 (88)
Illicit drug use in the past year	
Yes	8 (50)
Crack/cocaine	1 (6.3)
Ecstasy	1 (6.3)
Methamphetamine	1 (6.3)
Marijuana	7 (44)
Injection drugs	1 (6.3)
No	8 (50)
Past incarcerations in lifetime	
Yes	2 (13)
No	14 (88)
Years in which participants first tested positive for HIV	
1985–2000	3 (19)
2001–2016	13 (81)
Visited a healthcare worker for HIV-related medical care within 3 months of diagnosis?	
Yes	15 (94)
No	1 (6.3)
Visited a healthcare worker for HIV-related medical care in the past year	
Yes	16 (100)
No	1 (0)
Current trusting relationship with healthcare provider	
Yes	15 (94)
No	1 (6.3)
Number of missed clinic appointments in the past year	
0	11 (69)
1	1 (6.3)
2	2 (12)
≥ 3	2 (12)
Currently taking antiretroviral medications	
Yes	15 (94)
No	1 (6.3)
Viral load	
Undetectable (< 50 copies/mL)	11 (69)
≤ 75 copies/mL	3 (19)
Unknown	2 (12)

The years in which participants first tested positive for HIV span from 1985 to 2016 with the majority (81%) of participants diagnosed after the year 2000. In terms of linkage to care, almost all participants (94%) visited a healthcare worker for HIV-related medical care within the 3 months of their HIV diagnosis and reported having a current trusting relationship with their healthcare provider. Over two-thirds (69%) of participants stated that they had not missed a single clinic appointment in the past year. Almost all (94%) were currently taking anti-retroviral medications. In terms of viral load suppression, 69% of participants (*n* = 11) stated that their last viral load was undetectable.

### Qualitative findings

Three major themes around the HIV diagnosis experience arose from the interviews: (1) Trauma resulting from HIV diagnosis experience, (2) Lack of patient-centeredness in the delivery and context of HIV diagnosis, and (3) Acceptance of HIV diagnosis over time. In addition to the results below, Table 2 summarizes themes and subthemes, and provides additional representative quotes from the in-depth interviews.

**Table 2:** HIV Diagnosis Experience Themes

Themes	Subthemes	Theme description	Additional Representative Quotes
Diagnosis trauma	Direct trauma	Refers to perception of HIV as a fatal diagnosis, anticipated loss of life goals and dreams.	“When I first got diagnosed, I thought it was a death sentence … I was shocked, disappointed with myself, disappointed with the person that gave it to me, depressed, and that’s pretty much it.” (Participant 5, age 40s, diagnosed 2010s) “I was overwhelmed, but I didn’t cry … there was so much going wrong in my life it was just another slap in the face … It was so sterile, so detached from emotion. It was like I would have a year left to live” (Participant 10, age 30s, diagnosed 2010s) “I cried, I thought my life was over, I was like I’m not gonna be able to have any kids, if I tell my parents about this they’re gonna go absolutely nuts … after I found out I was homeless, I had to go into the SRO system … Once I got into my apartment I pretty much was bound (there), stopped hanging out with my friends, stopped going to school, didn’t even go grocery shopping first couple of months, just smoking weed all the time …. I probably missed a lot of appointments, I didn’t really deal with it (Participant 7, age 20s, diagnosed 2010s) The struggle became even more harder because I had other things that I had to tackle, but that’s what was really the difficult—was the anxiety and the depression, that—it’s just—it’s hard to even explain that mentally, if you have a weak mind and you’re in a weak place, it’s easy for the devil to kind of play on those feelings, those emotions. You’re not suicidal—and I’m not a suicidal person, but I did try to commit suicide when I was with my ex, the ex who gave it to me. I did try to commit suicide while I was with him and we were living together, but again, I’m not a suicidal person. Again, that was one of those situations that was—I was dealing with a mental health situation.” (Participant 13, age 30s, diagnosed 2000s)
	Vicarious trauma	Indicates being affected by someone else with HIV being treated poorly or dying. The experiences of others may be witnessed directly or learned about through word of mouth. Usually refers to a family member or someone else emotionally close to the participant.	My grandpa’s brother was gay and died in the 90s from HIV. That was kind of our introduction to it, if you will. Because we grew up in a very religious family, and we didn’t really know too many gay people … a year or so before he died, we were all at my grandpa’s house, and he came over. This was after he’d been diagnosed with HIV, and everyone knew he had HIV. They came over, and they put all the kids in a different house, because my grandpa lived next to his sister. They took all the kids over to his sister’s house, and the adults stayed over at my grandpa’s house, so they could visit with him, but they didn’t want the kids around, because they didn’t want him to infect the kids (Participant 14, age 20s, diagnosed 2010s)
	HIV-related and intersectional stigma	Diagnosis added shame to the burden of intersecting stigmas	“My culture is something that is anathema to me … I don’t feel comfortable being who I am having not been able to choose. If I could go back again I would choose something else. It’s a horrible statement because it contradicts my overwhelming sense of pride in being different and unique … because I’ve had so much plight in my life in having to deal with so many obstacles that come from being black, I actually left the country for a number of years as some relief, escape … When I came back, the reverse culture shock was quite [] … being objectified or ostracized solely based on the color of your skin is literally insufferable. I don’t know how we survived for so long with the things that we have to deal with … a lot of oppression has caused me to take on behaviors of the oppress (or) and I tend to discriminate against my own kind. I think I deal with a lot of [homophobia] from black people and a lot of [racism] from gay people and a lot of my strongest fears and most scathing situations have come from other gay black men. [Being positive] is just not something I’m proud of … I don’t want to have that be another object or label that’s put on the already long list of labels. I have to deal with being a minority in several different ways … I haven’t told my family and the reason behind that is my mother, when she found out I was gay, said, ‘I don’t care just don’t get AIDS.’” (Participant 1, age 30s, diagnosed 2000s)
Lack of patient-centeredness	Healthcare system environment	Considers the atmosphere in the clinic or medical office: physical setting (comfortable or not) treatment by staff (welcoming or not, attentive or not), protocols for delivering care	“He was a general practitioner. He wasn’t my doctor. I had no relationship with him so he sent me to a clinic in Newark … there were benches lined up against the wall and people were waiting to be called in … they basically told me to go in peace … There wasn’t any kind of personal relationship. There was really not much else. I was a number.” (Participant 8, age 50s, diagnosed 1990s)
	Medical provider	Includes provider affect, experience, identity. Includes provider’s delivery of the diagnosis (callous, sensitive), level of engagement (attentive or not, willing to answer questions or not)	“It was a bit strange … there wasn’t taking by the hand, you have it. It was more like you have it. This is it, so you need to be on medication. They were pretty much straightforward about it. There was no beating around the bush. There was no pity party … They walked out the door and left [me] in a room, alone, by myself, I guess, to absorb this … I was a bit taken aback, so this is how it is when you get infected. This is how you’ve been delivered the message. It’s thrown into your face” (Participant 16, age 40s, diagnosed 2010s)
Acceptance of HIV diagnosis	Disclosure or sharing of diagnosis	Considers length of time before disclosure, with whom the participant shared diagnosis, reactions of those told, factors considered in the decision to disclose	“I feel like when you’re being authentically yourself and you’re being honest with people about it, and you let go of that mentality of feeling like you’re a stigma … Knowing that, for me, is that I’m always aware that if these people are as open-minded as I think they are or expect them to be, then they’re going to take what I’m saying at face value, and they’re gonna take my truth, and they’re going to absorb it and respect that and respect me.. That’s it.” (Participant 13, age 30s, diagnosed 2000s)
	Social support	Includes degree of comfort with HIV in social or family networks, nature of interpersonal relationships and emotional ties	“I don’t know adult life without HIV because it happened at 21 … I still struggle sometimes with the stigma because it’s coming back. It used to be really bad in the beginning and then when I moved to New York, people … were so open about it that it actually liberated me as far as the stigma was concerned.” (Participant 2, age 50s, diagnosed 1980s)
	Self-motivation/Personal growth	Describes action taken to learn more about HIV, via research or speaking with friends or providers. The additional information created an empowered mindset to view HIV differently from at the time of diagnosis.	“Before I was diagnosed honestly I believe my HIV is my karma because they say you never wish anything on somebody else that’s wrong with yourself. When I was younger I talked about people with AIDS and I honestly thought it was a death sentence. You did this. I thought I would never catch it. Like, I could never catch this. Once catching it, I read up on the information about it, the disease, this, that … I said okay it’s not a death sentence cuz medicine is not where it was … It’s totally different now. It’s manageable now. You can’t be ignorant to being HIV positive. It’s not a death sentence. If you’re taking care of yourself, it can be managed.” (Participant 15, age 30s, diagnosed 2000s) “Just a few years ago it was a death sentence. In 10 years, you’re gonna die. In five, you’re gonna die and so you gotta take this. You’re gonna lose weight. You’re gonna get skinny … Now people are living with it for 20 years” (Participant 11, age 50s, diagnosed 2010s) “My therapist was good because she let me know that it’s not a death sentence what I had, so I felt like it wasn’t the end of the world for me … I have a more positive outlook on life itself, being HIV positive is not a death sentence, you know.” (Participant 5, age 40s, diagnosed 2010s)

### Diagnosis trauma

For most participants, being diagnosed with HIV was emotionally crippling and put life on hold. For some, their fears were shaped by what they had witnessed of friends’ and loved ones’ experiences with the disease and by the prospect of positive HIV status adding to the intersectional perceived stigma that accompanies black MSM identity.

#### Direct trauma

The majority of participants described immediate thoughts about death after receiving an HIV diagnosis. This response was consistent across age and date of diagnosis, beginning with those diagnosed in the 1980s and 1990s before the development of effective antiretroviral therapy. Participant 8 recalled being stunned at his diagnosis, and feeling his life was over as he saw friends becoming sicker while taking zidovudine, or AZT, the only antiretroviral medication available at the time.

“I remember thinking, ‘Oh my God this is now going to be my life, this is what I have to look forward to? It took a little time to grieve, I felt sad but I immediately got plugged into different groups...I was living at home, ashamed, I come from a family where we talk but not really – I didn’t want to be judged, I didn’t want to worry my mother. The movie Philadelphia just came out and there was a part where Denzel picks up his daughter and I remember my niece was just born and I thought “Oh my God, I’m never going to see her grow up.” (Participant 8, age 50s, diagnosed 1990s)

For some people diagnosed during the earlier years of the epidemic, the diagnosis was in fact presented as a death sentence, leading to engagement in risky behavior. Participant 2 described receiving his HIV diagnosis as a death sentence when he was a young man in the Navy, leading to elevated risk behavior.

“I was told I had six months to live so I was devastated and angry, confused, unsure, you know in disbelief, started acting out sexually, drinking more and doing recreational drugs, became a little more reckless because I felt like what did I have to lose … I had planned to get married. I had planned to have children. I had planned to go to Japan, to have these beautiful bi-racial children and when they told me that, all my plans ended. You’re getting ready to die. Don’t make plans, just take it day by day. Do whatever you wanna do. When they told me, it was a death sentence. I was there right at the beginning. There were no options … I still wanted to dream … I decided my life was over. I was still waiting to die. Six months had passed, but I was still waiting. Any moment it’s gonna happen.” (Participant 2, age 50s, diagnosed 1980s)

Despite the advent of ART in the late 1990s, participant 16, diagnosed nearly three decades after participant 2 (above), similarly saw HIV as a death sentence and engaged in riskier behavior because of it. Although Participant 16 described not being surprised by his HIV diagnosis, he pointed to the mental toll and isolation that occurred as a result. This posture of self-defense has been a part of his life ever since.

“I felt alone. I felt like someone slapped me in the face, physically, without the pain. Yes, I felt cold. I did feel alone. I felt as if I did something wrong. I felt also maybe my life is going spiraling down … I did drink more significantly than I ever have. I used (marijuana). I did not say much to others. I became angry … at first I saw it as a death sentence. Only reason being is research was not significant enough to make me feel comfortable there was a possibility of there being a cure … I thought maybe yes, there’s a possibility of a life-threatening (diagnosis).” (Participant 16, age 40s, diagnosed 2010s)

Another important subtheme was feeling that the diagnosis thwarted life goals and dreams, as Participant 13 explains. As an artist and a dancer, he described a celebratory mood going to multiple gay pride festivities in NYC the week prior to getting an HIV test, and then feeling numb after receiving his diagnosis.

“When he points and I see it says that I’m HIV positive, I’m—I’m just stuck for a minute because I’m—I couldn’t believe this was my reality at that moment, and I didn’t say anything for maybe—it was probably maybe three minutes, but it felt like eternity, and I just didn’t say anything, and he kept—he was calling me … All these things started flashing before me, like wow—like all the things that I wanted to do—me pursuing my passion to sing and to dance and act, and I was just, ‘Am I ever gonna be able to do any of this stuff?’” (Participant 13, age 30s, diagnosed 2000s)

Participant 11 discussed the same sense of loss and resignation after receiving the one diagnosis he had always been dreading.

“First thing that came out of my mouth was ‘Oh my God I’m not gonna be able to have children’ … I kind of knew it in a way, it was like a ticking time bomb until it happened, [then] it felt like ‘Okay the pressure’s gone, things are a little bit more mellow.’” (Participant 11, age 50s, diagnosed 2010s)

#### Vicarious trauma

Participants also recalled that their association between an HIV diagnosis and death resulted from ingrained beliefs or specific familial or cultural experiences. Participant 2 described seeing multiple other black gay men dying from HIV during the early years of the epidemic. He recalled the trauma of seeing people close to him succumb to the disease.

“It’s like receiving a cancer diagnosis … when things are terminal people tend to whisper about them or especially with HIV or AIDS. You were a leper. You didn’t want to be stigmatized, you didn’t wanna be discriminated against. When I was diagnosed, what we saw as Black gay men was one week Peter was here, the next week Peter wasn’t here. What happened to Peter? He went home. That was a very common phrase in the 80s and 90s … usually meant he went home to die.” (Participant 2, age 50s, diagnosed 1980s)

For Participant 7, the trauma provoked by his diagnosis was shaped by his memory of his aunt’s experience, not only because of her death but because his family denied her dignity because of their cultural perspectives.

“I come from a mixed household, my mom’s southern black American and my dad’s Jamaican … On my mom’s side it’s a lot more brutal, I don’t want them to know at all. Cuz I had an aunt who died of AIDS and when she died, it was just a lot of gossip about who she was sleeping with, how she was living her life. I was nine … it was a lot of horrible talk about gay people, even though the man she was dating didn’t identify as gay. Because she died of AIDS, it was this assumption that he was gay or doing drugs … I’m from the South, from Florida. The South can be very homophobic. Me growing up hearing about HIV and AIDS, it was always coined with gay men. We actually had a person in our family who died from full-blown AIDS. The only reason why she died is because she didn’t tell anybody.” (Participant 7, age 20s, diagnosed 2010s)

#### HIV-related and intersectional stigma

Participant 11 described the overlapping stigma of being black, Dominican, gay and HIV-positive, which included cultural judgment.

“When I was younger it didn’t mean that much to me but to know what it means to be black and Latino and to be a minority, it means a lot now … Both my grandmothers, they got scared … I feel like they equate gay to HIV and then death. That’s how it works … My father’s side of the family is old-school Dominican … for them, it was like, it is a death sentence. (Participant 11, age 50s, diagnosed 2010s)

Participant 2 was a healthcare worker when he contracted HIV in the 1980s. After his diagnosis, no longer allowed to work in the clinic, he was transferred to an office doing paperwork.

“I was upset because I had joined to do medicine and they were just so fearful back then that you would contaminate or infect other people they just took me out of direct patient care. I was going through my emotions … being told that I had six months to live and then having my career snatched from me, I was going through a lot. I was 21. There were no medications. There were no protocols for drawing labs or there was no viral load. There was none of that … so we didn’t have any follow-up. I had a discharge physical … I felt that he [the physician] was frustrated because he didn’t have anything to offer me … He couldn’t provide me with hope or say next month this new medication is coming out. There was none of that.” (Participant 2, age 50s, diagnosed 1980s)

Participant 2 felt doubly betrayed by the healthcare system when he received his diagnosis: his clinic employer treated him as a pariah due to his HIV and the diagnosing physician was unable to provide the level of care he needed.

Although the majority of participants described feelings of shock and shame and fear of rejection, a couple of participants did not describe their diagnosis taking a large toll. They recounted feeling surprised by their HIV diagnosis; however, they immediately sought out help from friends, family, therapists and other resources.

“While I was pretty much astonished, I was, ‘Oh okay’ because I’ve had sexual relationships before and nothing happened so that’s why I felt that it couldn’t happen to me. I started trying to figure out different places I could get some help … I wasn’t super duper blown up or anything like that.” (Participant 5, age 40s, diagnosed 2010s)

“I actually had no response, and the doctor looked at me and said, ‘I just told you you’re HIV-positive.’ I had no response because I have a melancholy attitude, so I was like, okay all I have to do is make myself better and trying to make myself healthy … I was in Syracuse. I remember when she said, ‘You have HIV.’ I said, ‘Okay,’ saying to myself I have to deal with it. I’ve been dealing with it all these years, and it’s been something that I have to do to keep alive, that’s what I do. Take my medications. I just try to keep healthy.” (Participant 12, age 50s, diagnosed 1980s)

### Lack of patient-centeredness at time of diagnosis

Despite the majority of participants reporting trusting relationships with their current HIV provider, most described negative experiences in the way their HIV diagnosis was delivered. Regardless of age or date of diagnosis, they described anger and disappointment with how they were treated and with the language used to disclose their results to them. This persisted across age and date of diagnosis.

#### Healthcare system environment

Participant 15 described fragmented care, receiving his initial diagnosis after screening for blood donation, then was bounced between the health department and the prison system, expressing distrust of the physicians in each setting.

“I went to the Health department and I took the test and it came back inconclusive … I was in denial the whole time. I was 20 years old … I got locked up, I went to prison. When you go to prison, they give you the whole screen down of everything. They’re gonna give you every kinda test they can on you. When I got there, they said, ‘You’re HIV positive.’ I was like—I already knew, but they kept telling me that my bloodwork was fine, that I didn’t have to worry about meds. My viral load was high, but my CD4 was still high, so I just didn’t worry about it … In prison the doctors they honestly want to … force a pill down your throat, and let that be the end of it. You be their Guinea pig. I wasn’t with that. I said as long as my CD4 was high I should be good … I really just kept to myself in prison.” (Participant 15, age 30s, diagnosed 2000s)

Participant 7 discussed feeling that the response from the clinic staff where he was diagnosed did not meet the gravity of his status. He felt forced to deal with the diagnosis like the healthcare staff wanted him to rather than being allowed to have a natural reaction.

**“**I went to the clinic. They retested me. They retested me twice. I got emotional. When I was emotional, they were very surly about it, and dismissive about it. I didn’t like that. I didn’t know what to do, so I was just stuck in this place. I didn’t know anywhere else I could go … they didn’t make me feel comfortable at all. There was a protocol there but it just seemed like there was this route of HIV negative and HIV positive. As soon as I found out I was positive, there was this rabbit hole they were just trying to force me down, instead of just trying to reaffirm that everything was gonna be okay … it just felt very aggressive is what I would say … When I went to [another New York hospital] it didn’t feel inviting at all. They were more so worried about we need insurance. We can’t see you without insurance. They were more so worried about the political stuff versus me just coming here for care.” (Participant 7, age 20s, diagnosed 2010s)

#### Medical provider

It is not always possible to separate HIV-related stigma or indifference from perceived homophobia in the course of receiving a diagnosis from a provider who does not seem comfortable in the face-to-face interaction during diagnosis. Provider affect can influence patient care engagement decisions, as Participant 6 recalled.

“I was taking the physical, the insurance company Omaha Insurance for their life insurance policy. They draw blood and all that stuff, come to your home … fill out some paperwork. They contacted me, I’d say about a week-and-a-half later and told me that I should see my PCP cuz some results or whatever were abnormal. Then when I actually talked to the nurse who actually came to the house, she said that my blood results came up positive for HIV … I got a confirmatory test done. Of course, they denied me my insurance. Going to the doctor, after finding out what was going on and stuff, he explained a few things … with him I always felt a little bit uncomfortable talking to him about that. He didn’t seem … gay-friendly. He probably was, but I didn’t get it … He’s probably the reason why I chose not to get on medication.” (Participant 6, age 40s, diagnosed 2000s)

Participant 14, diagnosed most recently out of all participants, felt his provider at the time lacked empathy. The provider attempted to minimize the impact of his diagnosis given by explaining he had planned to prescribe medication regardless of the HIV test result, which felt disrespectful.

“At first I didn’t believe it … the guy said, ‘The good news is you don’t need PrEP because you’re already positive.’ I was just like—I was like no, you’re not talking to me like that. I didn’t believe it, cuz I was like I just got tested, and I tested negative … it was the lack of care and concern. Like who the fuck would say something like that? The good news is you don’t need PrEP because you’re positive. That’s good news? Oh. Where?... When I was treated with that, I was just completely like this is not your bedside manner. This is not how you present somebody with information like that. Yeah, I went through all the emotions, all the feels immediately. You have to be wrong, test me again. Rejection, and then anger of the delivery, and how he said it. Then, frustration, and then immediately sadness.” (Participant 14, age 20s, diagnosed 2010s)

As Participant 14 discovered, provider thoughtlessness can compound the distress caused by an HIV diagnosis. His experience also may point to a potential moral hazard of the HAART era: that providers accustomed to HIV as a manageable chronic condition may lose sight of the fact that patients may be less well informed and are at different stages socially and emotionally.

### Acceptance of HIV diagnosis over time

The majority of participants noted that over time they became accepting of their diagnosis and viewed HIV differently. For some men, comfort with their status increased as they learned more about treatment options and as they found supportive peers or providers. They were able to talk about their status with others and to think in terms of planning for longer life.

#### Disclosure

Participant 6 began educating his mother after gaining knowledge about living with HIV in the era of effective therapies.

“We were actually talking in reference to one of [my mom’s] former coworkers—they retired now—her son passed away from complications. I was trying to explain to her, at this day and age, people who pass with complications from HIV and AIDS and stuff like that is almost unheard of. More than likely, they hadn’t been taking care of themselves. Me, personally, I haven’t heard of anybody actually passing away from HIV and AIDS or whatever, since ‘99 or 2000 or something like that.” (Participant 6, age 40s, diagnosed 2000s)

Participant 8 illustrated how some black MSM have had a longer journey to acceptance. After describing the trauma and fear of never living to see important milestones in his life after being diagnosed in the early 1990s, he described the hope he feels living free of the constant fear of death.

It was a different world back then, people were dying, there was a lot of stigma, there was a lot of fear, a lot of judgment. I didn’t tell anybody for a long, long time … there’s still a lot of stigma, a lot of judgment, but the fact is people aren’t dying as much as they were back then. It’s manageable now. It’s no longer this thing that could possibly kill me. It’s a part of who I am now. Yes, it’s a discussion that I now have to have with partners forever, but the chances are that people will not reject me because of it.” (Participant 8, age 50s, diagnosed 1990s)

As Participant 8 implied, a social environment that facilitates disclosure also helps to reduce the risk of transmission.

#### Social support

Participant 11 explained that finding a community of people living with HIV helped him accept his diagnosis.

“In the beginning we all have that thing, that whole death sentence thing. I remember one of the nurses or somebody sayin’ HIV’s very different from what it used to be years ago. Like I said, that stigma. My thought of HIV, now I understand people see it as a chronic illness, I don’t see it like that. It’s not even a stressor in my life … Once I found out, met new people who were HIV positive, I learned from them and I was able to realize it isn’t a death sentence as long as you make sure to take my medications every night and be very honest with my doctors.” (Participant 11, age 50s, diagnosed 2010s)

Participant 7 talked about the importance of getting more medical information and gaining knowledge about HIV as the reason he reached a level of comfort with his diagnosis.

“It doesn’t really affect my social life and my livelihood anymore. It’s not so bad as when I first started … I honestly compare with diabetes sometimes … [The doctor] just gave me a lot of information. I asked him, ‘I went on the CDC page and they say people only live up to 22 years.’ He was like ‘Don’t listen to that.’ He referred me to some men that he has known that’s been HIV positive for decades. Once he reassured me that it’s not a death sentence, I’m going to live past 22 years, I don’t know, I just felt more comfortable.” (Participant 7, age 20s, diagnosed 2010s)

#### Personal growth (self-motivation)

For some older participants, diagnosed in the era before HAART, personal drive was key to living through a long period of darkness. Participant 8 expressed a sense of pride in having survived since his diagnosis in the 1990s.

“I am not ashamed of it anymore. In fact, I see it almost as a blessing in a sense. It was this horrible thing that happened and I could have succumbed to it. I could have let it drag me under but I chose to become empowered.” (Participant 8, age 50s, diagnosed 1990s)

Personal growth toward living comfortably with HIV could also be driven by positive healthcare experiences and putting faith in medical advancements.

“I made my appointments … a lot of my friends in the gay society have died of HIV, just cuz they didn’t wanna take care of themselves, didn’t wanna commit themselves to lifelong regimen of meds. I say I’m not gonna be another stereotype. I’m gonna take care of myself … I do believe that people are living longer with the virus due to all the medications and resources they have out there now. I really don’t look at it as a death sentence. You have to take care of yourself initially, do the right things, and you can live longer.” (Participant 4, age 50s, diagnosed 2000s)

For some participants, being HIV positive has helped them become more conscious about their overall health. Participant 14 described how integral HIV has been not only to his journey to better health, but to his identity.

“It’s so comfortable being positive. Yeah, I definitely think there is enough resources for me to stay undetectable at the moment. I’m really content with that. My health has been great. I think my health has been better, now that I’m positive than I was when I was negative. It’s because I actually pay attention to it. I would before, I would get tested every three and six months and blah, blah, blah. Really, actually, I would only go and get tested at clinics, and I wouldn’t go to checkups. I ended up in the hospital a lot more when I was negative and using [drugs], than I do now when I’m positive and sober … HIV is almost as important to me now as being a Black man, because it’s a thing that makes up who I am.” (Participant 14, age 20s, diagnosed 2010s)

By embracing living with HIV as part of his identity, in the same way he views his racial identity, Participant 14 inverted the model of intersectional stigma that contributes to diagnosis trauma and transformed it into intersectional pride.

It is important to point out that accepting the diagnosis does not negate the fact that living with HIV is a constant challenge. Participant 1 described the unique mode of sexual transmission that has made an HIV diagnosis challenging and stigmatizing.

“I struggle with (being positive) every single day … I just think because the correlation of the way that it is transmitted – going back to the “dirty little whore” theory – is that a lot of people still have that distinction. I think this is helpful but at the same time you can’t convince everyone that it’s like diabetes because you don’t get diabetes from sharing needles.” (Participant 1, age 30s, diagnosed 2000s)

Similarly, coming to terms with HIV diagnosis includes living with the possibility of one day becoming sick, as Participant 3 described.

“I’m not gonna say I don’t think about [getting sick], since I took care of a friend, I watched him go through that. I saw him at his worst and I’m like – think about that a lot like ‘This could be me or is that gonna be me or is it just a matter of time?’ All of this has been in the back of my mind, like eventually that day gonna come.’ I ain’t gonna say I don’t think about stuff like that. I do. It crossed my mind, but I don’t let it get me down.” (Participant 3, age 30s, diagnosed 2010s)

The narratives in our sample illustrate unique ways in which black MSM individually manage to overcome diagnostic trauma to remain successfully engaged in HIV care. Taken together, they also illustrate the common experiences of a diverse group of black MSM along the HIV care continuum after diagnosis.

## Discussion

Our study emphasizes that diagnosis is a time of particular vulnerability for black MSM along the HIV care continuum that warrants more attention. Our sample was homogenous in terms of race and sexual orientation yet diverse in terms of age and date of HIV diagnosis. Participants were diagnosed in a variety of settings, including clinic offices and workplace testing. They were also mostly retained in care and virally suppressed at the time of the interview. The perceived nature of the HIV diagnosis experience among this group has important implications for engagement in the care continuum and ongoing risk management. By exploring the impact of the diagnosis experience on the lives of a sample of black MSM living with HIV through in-depth interviews, we found that an HIV diagnosis may still carry significant personal trauma, rooted in lost life goals and anticipation of death, as well as vicarious trauma, reflected in the significant community-level suffering by both the black community and the gay community over the history of the epidemic. The role of trauma, defined as experience of an event physically or emotionally harmful or life-threatening with lasting adverse effects on the individual’s functioning and mental, physical, social, emotional, or spiritual well-being, warrants further exploration at diagnosis for black MSM [18]. For example, a recent study by Burnham et al. among HIV-positive gay and bisexual MSM explored how more trauma-related symptoms indexed at diagnosis and internalized stigma lead to more HIV transmission risk behaviors [19]. Despite groundbreaking biomedical advancements in HIV treatment and the shifting national understanding of HIV as a chronic illness, we found that there is still a particular association between an HIV diagnosis and a death sentence that has persisted across age differences. Thus, scientific discovery has not fully mitigated the psychological trauma of HIV for black MSM.

Some participants feared HIV as a terminal diagnosis. However, beyond the physical loss of life, participants pointed to the idea of a “social death” [20]. This term originated from the field of anthropology and refers to a group of people no longer being regarded as fully human by society [21]. The notion of “social death” captures our participants’ fear that becoming HIV-positive would mean losing one’s basic possibilities of human realization in society, such as having children or being able to fulfill life dreams. Additionally, this fear was grounded in HIV-related and intersectional stigma as a result of particular family experiences, such as watching a family member or friend die of HIV/AIDS and witnessing their ostracization and suffering as a result of cultural belief systems. This vicarious or intergenerational trauma had salience for many participants in the HIV diagnosis experience. Increased community awareness of advancements in HIV science may help to soften persistent patient perceptions about HIV diagnosis as a death sentence or loss of possibilities in life.

We also found that the majority of participants had HIV diagnosis experiences marked by a lack of patient-centeredness on the part of healthcare providers and in their interaction with the healthcare system. Given the historic mistreatment of racial and sexual minorities (and those at the intersection of those identities) by the healthcare establishment, this population is likely knowledgeable of the potential for further stigma, judgment, and discrimination prior to and during an HIV diagnosis [22]. It is possible that providers become indifferent to these contextual factors, particularly if they think of HIV as a chronic condition. Patient-centeredness refers to an approach that emphasizes patients’ cultural backgrounds, values, and belief systems. We found that specific negative interactions during diagnosis disclosure were easily recalled, in some cases decades later. One participant specifically described delaying care and treatment as a result. This points to critical deficiencies in the delivery of an HIV diagnosis to black MSM, specifically the failure to acknowledge associated trauma. A delivery of HIV diagnosis that is not patient-centered inflicts further trauma perpetrated by the very healthcare system upon which patients depend to maintain treatment adherence and viral suppression.

Interventions that accompany HIV diagnosis could focus on facilitating acceptance, helping recently diagnosed people educate their support members about advancements in treatment, and establishing ways to provide caregiver support and education. Programs may model an approach to HIV diagnosis for particularly vulnerable populations such as black MSM under trauma-informed care–an approach that recognizes existing race-based and sexual-orientation-based trauma and incorporates anticipation of internalized HIV stigma at the time of diagnosis [23].

Our understanding that the trauma of HIV diagnosis has persisted over time for black MSM has implications for implementation of HIV-related interventions across the HIV care continuum. For example, with the expansion of at-home testing for HIV and rapid ART initiation programs, there may be opportunities for innovative approaches that acknowledge these experiences of trauma. Furthermore, understanding the extent of trauma allows an opportunity for HIV provider education regarding the mechanics of diagnosis in the current era, as there may be a knowledge gap in terms of practitioners’ understanding of the particular impact of an HIV diagnosis on black MSM. Over time, healthcare providers may assume that most patients believe HIV is a chronic illness at diagnosis and may not know the appropriate emphasis to put on discussions of life expectancy and the social implications of an HIV diagnosis. Another potential intervention may involve linking newly diagnosed black MSM to HIV-positive coaches, older people living with HIV or survivors, who can speak to life after diagnosis and offer a point of contact, connection, and normalization.

Despite our findings of extensive trauma during HIV diagnosis, the group of black MSM included in our study showed resilience in the acceptance of their diagnosis over time. The quantitative results show that this sample is mostly adherent to medications, currently in care, and virally suppressed despite initial traumatic experiences. Each participant recounted their unique journey to remaining in care and living openly with HIV, such as Participant 8 who described initial fear of death upon diagnosis in the 1990s to HIV presently being fully integrated into his identity. Many sought providers and clinical spaces where they would feel most comfortable and remain engaged in care. They also showed significant post-traumatic growth, such as Participant 14, who expressed shock and anger at how his diagnosis (in the 2010s) was delivered but lives as proudly as a Black man as he does living with HIV. This post-traumatic growth may explain this group’s positive HIV care continuum outcomes [24, 25]. While many HIV-positive black MSM overcome the trauma of initial diagnosis, it is important to understand and replicate the aspects of care that drive this process of acceptance, which for our cohort included knowledge acquisition and patient-provider communication.

There are several limitations to this study. The cohort was small and recruited mostly from healthcare settings. In addition, as we sought to recruit from a variety of settings, HIV status was determined by self-report. Furthermore, their experiences represent largely one urban environment in NYC. Participants were also not diverse in terms of ethnicity or sexual orientation, and all reported sexual intercourse with another man in the previous 12 months, which may bias the sample to sexually active black MSM and limit generalizability. It is important not to generalize these results to all black MSM because the experiences of black MSM of immigrant experience or bisexual black MSM, for example, may differ based on their varied identities. Another limitation to this study is that participation in an in-depth interview risks selection bias given that participants who agree to take part in the study are more likely to be engaged in care. Indeed, nearly every member of our sample was on medication and reported good retention in care. Although it is important to hear from those black MSM who have been successful along the HIV care continuum, this assessment of the experience of HIV diagnosis may not be comprehensive. Future research can explore the diagnosis experiences of previously diagnosed HIV-positive black MSM who are not retained in care, which would provide deeper understanding of the impact of initial diagnosis on ongoing care and viral load suppression.

## Conclusions

In this small pilot study, our qualitative results signal that an HIV diagnosis experience possibly remains traumatic for black MSM even in the era of HAART, and they often perceive a lack of patient-centeredness in the delivery of a new diagnosis. This has persisted over time. In most cases, black MSM in our sample overcame this trauma due to self-motivation, social support and seeking out and fostering trusting relationships with their HIV provider and the healthcare system.

## Supplementary information

**Additional file 1.** CDUHR HIV Positive Black Men Who Have Sex with Men (MSM) Questionnaire. Quantitative survey capturing basic demographic information including age, race/ethnicity, sexual orientation, level of education, income, housing, employment, history of incarceration and health insurance status.

**Additional file 2.** Personal Interview Question Guide. Interview guide organized by the HIV care continuum and included in-depth questions about the diagnosis experience, barriers to linkage to and retention in care, barriers to medication initiation and adherence, and the role of racial, sexual and cultural identity in HIV care.

**Additional file 3.** COREQ Checklist. Checklist of COREQ guideline items included for this qualitative study.

## Data Availability

The data that support the findings of this study are not publicly available due to participant confidentiality restrictions but are available from the corresponding author on reasonable request.

## Abbreviations

NYC: New York City
MSM: Men who have sex with men
COREQ: Consolidated criteria for reporting qualitative research
ART: Antiretroviral therapy
