# Supplementary material for: “Like a ticking time bomb”: the persistence of trauma in the HIV diagnosis experience among black men who have sex with men in New York City
Source: BMC Public Health. 2020 Aug 17;20:1247. doi: 10.1186/s12889-020-09342-9 (PMC7433074; doi:10.1186/s12889-020-09342-9)
Supplement: Supplementary file 1 — Additional file 1. CDUHR HIV Positive Black Men Who Have Sex with Men (MSM) Questionnaire. Quantitative survey capturing basic demographic information including age, race/ethnicity, sexual orientation, level of education, income, housing, employment, history of incarceration and health insurance status. [file 12889_2020_9342_MOESM1_ESM.docx]

**CDUHR HIV Positive Black Men Who Have Sex With Men (MSM) Questionnaire**

**We are interested in learning more about the experiences of HIV+ black MSM in New York City. This questionnaire is meant to understand better how to deliver the best HIV care and treatment to this community. If you have any questions or concerns, please contact Dr. Ofole Mgbako at** [**ofole.mgbako@nyumc.org**](mailto:ofole.mgbako@nyumc.org)**.**

***Basic Demographics***

Study ID_______

Date of birth (mm/dd/yyyy)? __________________

1) Are you a US citizen? [ ] Yes [ ] No

2) What is your race/ethnicity (check all that apply)?

[ ] Black/African American

[ ] Hispanic or Latino

[ ] Native American/Alaskan Native

[ ] Asian

[ ] Native Hawaiian or Other Pacific Islander

[ ] White

[ ] Mixed

[ ] Black African

[ ] Black Caribbean

[ ] Unknown

[ ] Other (specify): _________________________

3) In the last 12 months, have you had sexual intercourse (oral, anal) sex with a man?

If yes, with how many men have you had sex in the last 12 months? ________

*If patient meets criteria based on ethnicity and other established inclusion criteria, move on to next question*

4) Which borough of New York City do you currently live in?

[ ] Bronx

[ ] Brooklyn

[ ] Queens

[ ] Long Island

[ ] Manhattan

[ ] Other_________

5) What is your sexual orientation (check all that apply)?

[ ] Heterosexual/straight

[ ] Bisexual

[ ] Lesbian

[ ] Gay/homosexual

[ ] Transgender (female to male)

[ ] Other:_______________

6) What is your highest level of education?

[ ] No school

[ ] Elementary school

[ ] Some high school

[ ] High school diploma or equivalent (GED)

[ ] Vocation/technical school

[ ] Some college

[ ] Associate degree

[ ] Bachelor’s degree

[ ] Doctoral degree

[ ] Professional degree (MD, JD, etc.)

7) What is your current employment status?

[ ] Employed for wages

[ ] Self-employed

[ ] Unemployed and looking

[ ] Unemployed and not currently looking

[ ] Homemaker

[ ] Student

[ ] Military

[ ] Retired

[ ] Unable to work

8) What is your current housing situation?

[ ] I have current housing [ ] I am currently homeless

9) Have you been homeless within the last 12 months?

[ ] Yes  [ ] No

10) What is your current income?

[ ] <$20,000

[ ] $20-40,000

[ ] $40-60,000

[ ] $60-80,000

[ ] $80-100,000

[ ] >$100,000

[ ] Rather not say

11) Do you currently have health insurance?

[ ] Yes [ ] No [ ] Don’t know

12) What is your relationship status?

[ ] Married   [ ] Divorced   [ ]  Single    [ ] Partnered   [ ] Widowed

13) Have you ever been incarcerated?

[ ] Yes [ ] No

***HIV information***

1) What month and year did you first test positive for HIV (mm/yyyy)? ____________

2) How did you contract HIV?

[ ] Sexual relationship  [ ] Mother-to-child  [ ] Sharing needles  [ ] Unknown

3) What was your most recent CD4 count? ___________   [ ] Unknown

***Sexual Practices/Drug-Related Risk***

1) Have you been sexually active within last 12 months? [ ] Yes [ ] No

2) In the last 12 months, have you had sexual intercourse (oral, vaginal, anal) sex with a women? [ ] Yes [ ] No

If yes, with how many women have you had sex in the last 12 months? ________

3) Was your last sexual partner (check all that apply)…

[ ] HIV-positive

[ ] HIV-negative

[ ] Black/African American

[ ] Hispanic or Latino

[ ] Native American/Alaskan Native

[ ] Asian

[ ] Native Hawaiian or Other Pacific Islander

[ ] White

[ ] Mixed

[ ] Black African

[ ] Black Caribbean

[ ] Unknown

[ ] Other (specify): _________________________

4) Have you been diagnosed with another sexually transmitted infection (STI) in the past 12 months?

[ ] Yes  [ ] No

a) If so, which STI?

[ ] Syphilis

[ ] Herpesvirus

[ ] HPV

[ ] Chlamydia

[ ] Gonorrhea

[ ] Other

5) Did you use illicit drug use in the last 12 months?

[ ] Yes [ ] No

a) If so**,**which ones?

[ ] Crack/cocaine

[ ] Heroin

[ ] Ecstasy

[ ] Methamphetamine

[ ] Marijuana

[ ] Other___________________

6) Did you use injection drugs in the last 12 months?

[ ] Yes [ ] No

***Linkage to HIV Care***

1) Did you visit a doctor or health care worker for HIV medical care within 3 months of your HIV diagnosis? [ ] Yes [ ] No [ ] Don’t know

2) If no, what was the main reason you didn’t go to a doctor or other health care worker for HIV medical care within 3 months of testing positive for HIV (check all that apply)?

[ ] I felt fine

[ ] my initial CD4 count and viral load were good

[ ] I didn’t believe the test result

[ ] I didn't want to think about being HIV positive

[ ] I didn’t have enough money or health insurance

[ ] I had other responsibilities such as child care or work

[ ] I was homelessness

[ ] I was actively drinking or using drugs

[ ] I felt too sick

[ ] I forgot

[ ] I scheduled but missed appointments

[ ] I moved or I was out of town

[ ] I was unable to get transportation

[ ] I didn’t know where to go

[ ] I couldn’t find the right HIV health care provider

[ ] I was unable to get earlier appointment

[ ] I was unaware of recommendation to enter care within 3 months

[ ] Other_____________________________________

[ ] Don’t know

***Retention in HIV Care***

1) When was the most recent visit to a health care worker for HIV medical care before today (month/year)? _____________

2) Do you have a trusting relationship with your health care provider?

[ ] Yes [ ] No [ ] Sometimes [ ] Don’t know

3) How many clinic appointments have you missed in the past year?

[ ] 0 [ ] 1 [ ] 2 [ ] >3

***Maintenance on antiretroviral (ARV) therapy***

1) Are you currently taking ARV medication? [ ] Yes [ ] No

a) If no, what is the main reason you are not taking ARV medication (check all that apply)?

[ ] I don’t want to

[ ] my doctor told me to delay or stop treatment

[ ] I don’t like the side effects

[ ] I am too depressed or overwhelmed

[ ] I am currently drinking or using drugs

[ ] I have money or insurance problems

[ ] I am homeless

[ ] I am taking alternative or complementary medicines

[ ] Other______________________________

[ ] Don’t know

2) In what month and year did you begin taking ARV medication? _______

3) How many times have you switched ARV medication?

[ ] Never [ ] 1 [ ] 2 [ ] >3

***Viral Suppression***

1) What was your most recent viral load? [ ] ______  [ ] Undetectable   [ ] Unknown

3) How many times have you been hospitalized in the past year due to an HIV-related illness?

[ ] 0 [ ] 1 [ ] 2 [ ] >3

**Thank you very much for your time. We appreciate your participation in this study on the experiences of HIV+ black men which help us better identify potential solutions for addressing the epidemic among this group in New York City.**
