# Supplementary material for: “Like a ticking time bomb”: the persistence of trauma in the HIV diagnosis experience among black men who have sex with men in New York City
Source: BMC Public Health. 2020 Aug 17;20:1247. doi: 10.1186/s12889-020-09342-9 (PMC7433074; doi:10.1186/s12889-020-09342-9)
Supplement: Supplementary file 2 — Additional file 2. Personal Interview Question Guide. Interview guide organized by the HIV care continuum and included in-depth questions about the diagnosis experience, barriers to linkage to and retention in care, barriers to medication initiation and adherence, and the role of racial, sexual and cultural identity in HIV care. [file 12889_2020_9342_MOESM2_ESM.doc]

**Personal Interview Question Guide**

Interviewer: Today we will be discussing your experiences with HIV. We will be discussing when you were diagnosed, as well as different challenges to starting treatment and staying on treatment. We will also discuss what role, if any, your cultural background has played in making it easier or harder to live with HIV. This interview will take 2 hours at the most. Feel free not to answer any question, which makes you feel uncomfortable. Feel free to ask me to repeat questions at any times. Please remember if at any point you would no longer like to take part in the study, it is your right to do so. We will be audio-recording the interview today, and I want to reassure you that your name and any other identifiers will not be included in the transcript. Do you have any questions? If not, we will get started.

**Barriers to linkage to care for HIV-positive black MSM**

Where were you when you were first diagnosed with HIV?

When were you first diagnosed with HIV?

What was your reaction when you first learned you were HIV positive?

How did you initially deal with your diagnosis?

Did you share your diagnosis with anyone? Family? Friends? Why or why not?

How long did it take you to see a doctor after your HIV diagnosis?

What was your experience during your first doctor visit after diagnosis?

How long did it take until you began treatment? Why?

How does your view of HIV after your diagnosis differ than prior? Some people may view HIV as a chronic illness, while others may view it as a death sentence, or something in between.

**Barriers to retention in care for HIV-positive black MSM**

After your first visit to a doctor after diagnosis, did you follow up as directed?

Does HIV impact your daily life? If so, how does it impact your daily life?

Does your social environment - the neighborhood in which you live, your housing situation - affect how often you make your doctor appointments?

Do you openly discuss being HIV positive with people in your life? Why? Why not?

Do you believe you have a support network (e.g., family, friends) that helps you deal with having HIV?

What role, if any, does your race play in discussing your diagnosis with your support network such as your family/friends/significant others?

What role, if any, does your race play in discussing your diagnosis with your doctor?

What role, if any, does your sexual orientation play in discussing your diagnosis with your family/friends/significant others?

What role, if any, does your sexual orientation play in discussing your diagnosis with your doctor?

Have you ever been very sick from your HIV?

How has being HIV positive changed your family relationships or friendships?

How has being HIV positive changed your romantic relationships?

How has being HIV positive changed your relationships with sexual partner or partners?

Describe your experiences with health care providers since your diagnosis?

Was it easy to communicate with your healthcare provider about your diagnosis? About your sexual identity?

Did you ever feel your healthcare provider made certain assumptions about you?

**Barriers to medication adherence/viral suppression for HIV-positive black MSM**

What are the challenges to taking your antiretroviral medication?

Have you ever been very sick from taking your antiretroviral medication?

Does your social environment - the neighborhood in which you live, your housing situation - affect how often you take your medication?

Do you think it is important to know what your CD4 count and viral load are?

Do you have a discussion about being HIV positive before sex?

Do you think there are enough resources available to treat your HIV?

What do you think of pre-exposure prophylaxis or PrEP?

**Cultural Identity**

How important is your culture to you?

How important is your ethnic identity to you?

Do you think your ethnic or cultural identity affects your ability to talk about your diagnosis with your provider?

Do you think your ethnic or cultural identity affects your ability to talk about your sexual identity with your provider?

Do you think the race or ethnicity of your healthcare provider is important?

Do you think the sexual orientation of your healthcare provider is important?

**Perspectives on solutions**

What do you believe needs to be done to decrease rates of HIV infection among black men?

What can be done to get more HIV positive black gay, bisexual and other men into care?

What can be done to maintain HIV positive black gay, bisexual and other men in care?

What can be done to ensure HIV positive black gay, bisexual and other men take their medication?

What can specifically be done to get more HIV positive gay, bisexual and other [insert black Latino, black American, black African, black Caribbean or biracial] men into care?

*MSM = men who have sex with men
